# Prevalence of intratubular germ cell neoplasia in cryptorchid testes of infertile men

**Published:** 2013-04

**Authors:** Fatemeh Pourkeramati, Haleh Soltanghoraee, Naser Amirjannati, Mohammad Mehdi Akhondi, Hamid Reza Reza Khorram Khorshid

**Affiliations:** 1 *Reproductive Biotechnology Research Center, Avicenna Research Institute (ACECR), Tehran, Iran.*; 2 *Genetic Research Center, University of Social Welfare and Rehabilitation Science, Tehran, Iran.*

**Keywords:** *Cryptorchidism*, *Germ cell neoplasm*, *Placental like acid phosphatase*

## Abstract

**Background:** Cryptorchidism is a common malformation in neonates; surgery or medical treatments are applied during childhood. Untreated cryptorchid testes are in the risk of intratubular germ cell neoplasia (IGCN) and consequently invasive testicular tumors which could be shown by immunohistochemistry staining for placental like acid phosphatase (PLAP) marker.

**Objective:** We designed this study to know the prevalence of IGCN in untreated cryptorchid testes of infertile men, in our infertility center as a refferal center.

**Materials and Methods:** In this cross-sectional study we assessed H&E slides of testicular samples of 13 adult infertile patients with impalpable intra-abdominal testes seeking infertility treatment; then we stained them by PLAP marker.

**Results:** Three (23.08%) samples were positive for PLAP marker means presence of IGCN in testis. One of them showed seminoma besides IGCN.

**Conclusion:** According to the results of this study and the fact that there are adult untreated cryptorchid patients in our country yet, it is suggested to pay more attention in clinical examination, assessment and follow up of such patients for malignancy screening.

## Introduction

Cryptorchidism (undescended testis) is the most common congenital malformation in newborn boys (about 3% of full-term infants).Testis may be located between the abdominal cavity and immediately external to the scrotum, unilaterally or bilaterally. From all undescended testes seen after birth, mostly descend normally within a few months. Only 1% with a persisting cryptorchid condition needs for medical or surgical treatments (1).

Intratubular germ cell neoplasia (IGCN) consists of enlarged spermatogonial cells with clear large cytoplasm, irregular nuclear borders, hyperchromatic and prominent nucleoli. IGCN is the precursor of invasive germ cell tumors. Immunohistochemistry staining for placental like acid phosphatase (PLAP) shows positive results in a high percentage of IGCN, while it is almost always negative in non-neoplastic spermatogonial cells (2). 

The relationship of cryptorchid testis to its late malignant transformation has been discussed in many studies. There is a risk of 30 to 50 times for malignancy occurring in an undescended testis than in a normally descended testis (3). Besides it seems that the time of orchidopexy is important in the risk of malignancy. Recent data suggest that risk of malignant degeneration may be 2-6 times higher in men underwent orchidopexy after puberty compared with those having surgery at an earlier ages (<10 years) (4, 5). Orchidopexy not only decreases the rate of malignancy but also exposes testes to be examined (6). 

In some references orchiectomy is the best suggestion for post-pubertal men up to age 50 years. Because these gonads have poor fertility potential and increased risk of malignancy, whereas non-operative treatment is recommended in men older than 50 years because there is no sufficient studies to define their cancer risk (7).

Yet the orchidectomy is a challenging issue in these patients, especially in infertile men. There is no previous study in our country to show the rate of malignancy of cryptorchid testes of adult patients. There are two reports in other countries showing the prevalence of malignancy about 4% (8, 9). This study performed to reveal the importance of follow up of infertile cryptorchid men, not only for treatment of their infertility but also for screening for IGCN.

## Materials and methods

**Figure 1:**
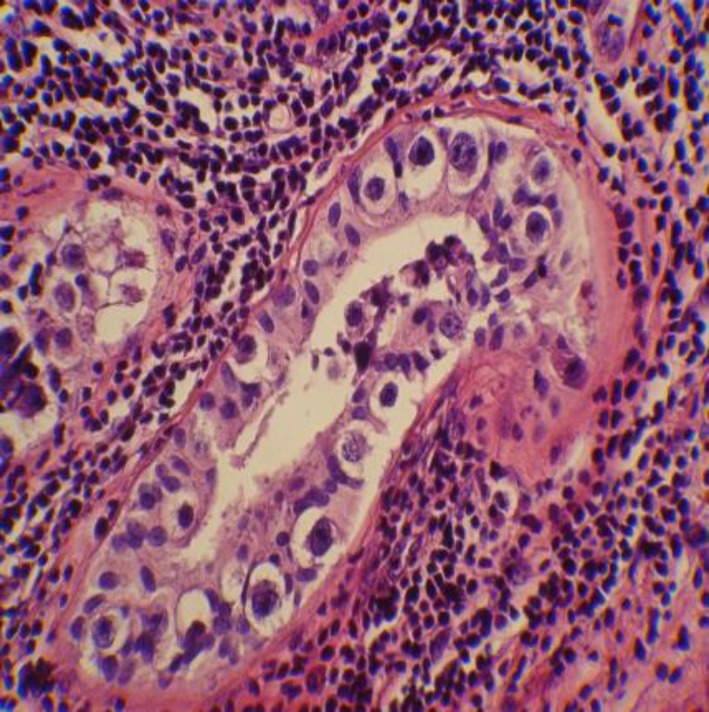
ICGN in H&E slide. These are cells with large clear cytoplasm, chromatin clumps in nuclei, irregular nuclear borders and atypical cellular shapes lined on tubular basement membrane

In referral Andrology Clinic of Avicenna Infertility Center, from 2004 to 2010, 13 infertile azoospermic patients were found for whom unilateral laparascopic orchidectomy due to unpalpable intra-abdominal testis had done. This cross-sectional study was approved by Avicenna Ethics Committee, and a signed consent was given by all patients. Patients were adults with untreated cryptorchidism and no one had a history of a previous malignancy especially in testis. All the testes were obtained by laparoscopic procedure. The mean age of patients was 35.53 years (27-43 yrs). Testicular samples were fixed overnight in Bouin’s fixative, directly after orchidectomy and subsequently embedded in paraffin. Two tissue sections were prepared from each sample then stained with Heamatoxylin and Eosin (H&E) (Figure 1) and immunohistochemistry staining of placental alkaline phosphatase (PLAP), which is routinely used as an IGCN marker. 

The immunohistochemical staining was performed using a standard indirect peroxidase method. Briefly, the dewaxed and rehydrated sections were heated in a microwave oven in a 10 mmol/l citrate buffer, pH=6.0 to unmask the antigen. Subsequently, the sections were incubated with H_2_O_2_ to inhibit the endogenous peroxidase, followed by protein block to block unspecific binding sites (Novocastra kit, RE7140-k). 

**Figure 2:**
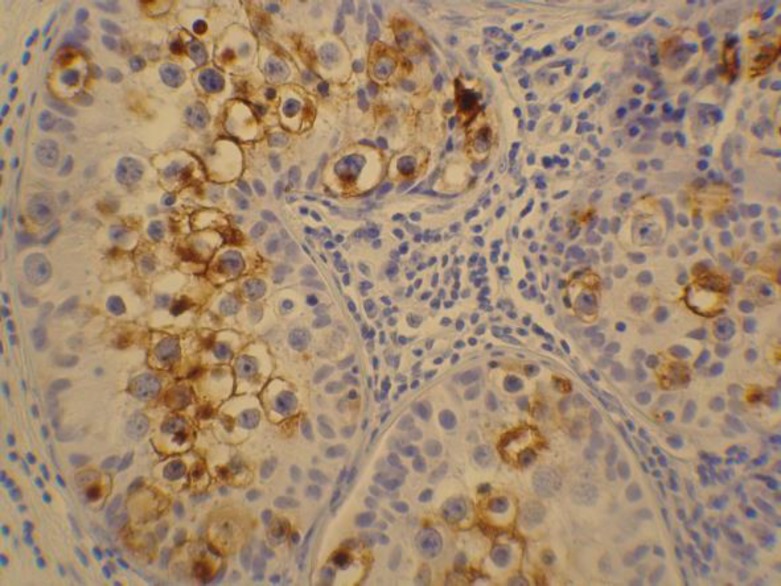
Positive cells (brown stained) for PLAP marker seen in these tubules proved the presence of IGCN in these tubules

The incubation with the primary PLAP mouse monoclonal antibody (Novocastra antibody, NCL-PLAP-8A9) diluted 1:20 was carried out for 40 minutes at room temperature. Subsequently, a HRP-anti-mouse link antibody was applied and visualized using DAB (diluted 1:20). Sections were then thoroughly washed. For negetive control, negetive tissue (tonsil) was used. Sections were counterstained with Mayer’s Haematoxylin to mark unstained nuclei. The sections were examined under light microscope by two pathologists (Figure 2).

**Statistical analysis**

We used Minitab 14 software and two proportions test to compare our results with other articles (H0: P1=P2).

## Results

Ten out of 13 assessed testes were left testis. The macroscopic evaluation and measurements revealed volume of testes between 2.4-24.75 ml. In H&E slides one of the samples showed seminoma besides IGCN and two slides had only IGCN. Immunohistochemistry for PLAP as a marker of IGCN proved the H&E findings, thus IGCN in examined testes corresponding to a prevalance of 23.08% while the invasive tumor rate was 7.6%. The mean volume of involved testes was 20.17 ml and the volume for testes without IGCN was 9.63 ml. Two out of three involved testes were left side. Age of patients having IGCN in their testis was between 32-37 years.

## Discussion

Modern analysis suggests that the rate of prior cryptorchidism in men with testicular cancer is around 5-10% (10, 11). While an accurate estimate of the lifetime risk of testicular cancer in a man without any risk factor has often been quoted to be 1 in 500 (12). As Skakkebaek indicated testicular dysgenesis syndrome (TDS) is related to the problems such as IGCN, cryptorchidism, hypospadias and low sperm counts, caused by prenatal testicular developmental disturbance (13, 14). 

Thus, in infertility clinics especially those encounter with chryptorchid patients, these risk factors should be consided. There are two studies like the present study in recent years, both in 2005: a study in Spain on 25 males from which one (4%) showed seminoma (8). In another study from Tunis 2 out of 81 (2.4%) cryptorchid patients older than the age 14, were found to have developed a testicular malignancy (9). 

The invasive tumor rate in our series was 7.6% which was the same as above mentioned articles (p-value 0.67, 0.75 respectively). On the other side the prevalence of 23.08% ICGN in this study is a considerable figure, which should be mentioned in clinical evaluation of infertile men. The problem of these patients could change from seeking fertility treatment to a general health risk. 

As a result of infrequency of these patients who had not undergone orchiopexy early in their life the expansion of samples was not practical. Thus we suggest more evaluation of such patients and considering them as high risk patients for malignancy beside treatment of infertility.

## Notes

*This article extracted from M.Sc. Thesis (Fatemeh Pourkeramati).*

## References

[1] Trussell JC, Lee PA (2004). The relationship of cryptorchidism to fertility. Curr Urol Rep.

[2] Montironi R (2002). Intratubular Germ Cell Neoplasia of the Testis: Testicular Intraepithelial Neoplasia. Eur Urol.

[3] Batata MA, Whitmore WFJ, Hilaris BS, Tokita N, Grabstald H (1976). Cancer of the undescended testis. Am J Roentgen.

[4] Pettersson A, Richiardi L, Nordenskjold A, Kaijser M, Akre O (2007). Age at surgery for undescended testis and risk of testicular cancer. New Eng J Med.

[5] Walsh TJ, Dall'Era MA, Croughan MS, Carroll PR, Turek PJ (2007). Prepubertal orchiopexy for cryptorchidism may be associated with lower risk of testicular cancer. J Urol.

[6] Chiba K, Ishikawa T, Yamaguchi K, Fujisawa M (2009). The efficacy of adult orchidopexy as a treatment of male infertility: our experience of 20 cases. Fertil Steril.

[7] Wood HM, Elder JS (2009). Cryptorchidism and testicular cancer: separating fact from fiction. J Urol.

[8] Granados EA, Loarca SEO (2005). Is necessary to practice orchiectomy in patients with post-puberal maldescended testes?. Actas Urol Esp.

[9] Ben Jeddou F, Ghozzi S, Rais NB (2005). [Cryptorchidism in adults. About 81 cases]. La Tunisie medicale.

[10] Kanto S, Hiramatsu M, Suzuki K, Ishidoya S, Saito H, Yamada S (2004). Risk factors in past histories and familial episodes related to development of testicular germ cell tumor. Int J Urol.

[11] Prener A, Engholm G, Jensen O (1996). Genital anomalies and risk for testicular cancer in Danish men. Epidemiology.

[12] Wood HM, Elder JS (2009). Cryptorchidism and Testicular Cancer: Separating Fact From Fiction. J Urol.

[13] Skakkebaek NE, Rajpert-De Meyts E, Main KM (2001). Testicular dysgenesis syndrome: an increasingly common developmental disorder with environmental aspects. Hum Reprod.

[14] Skakkebaek NE (2002). Carcinoma in situ of the testis: frequency and relationship to invasive germ cell tumours in infertile men. Histopathology.

